# A simple structure-based model for the prediction of HIV-1 co-receptor tropism

**DOI:** 10.1186/1756-0381-7-14

**Published:** 2014-08-01

**Authors:** Dominik Heider, Jan Nikolaj Dybowski, Christoph Wilms, Daniel Hoffmann

**Affiliations:** 1 Research Group Bioinformatics, Center of Medical Biotechnology, University of Duisburg-Essen, Universitaetsstr. 2, 45117 Essen, Germany

## Abstract

**Background:**

Human Immunodeficiency Virus 1 enters host cells through interaction of its V3 loop (which is part of the gp120 protein) with the host cell receptor CD4 and one of two co-receptors, namely CCR5 or CXCR4. Entry inhibitors binding the CCR5 co-receptor can prevent viral entry. As these drugs are only available for CCR5-using viruses, accurate prediction of this so-called co-receptor tropism is important in order to ensure an effective personalized therapy. With the development of next-generation sequencing technologies, it is now possible to sequence representative subpopulations of the viral quasispecies.

**Results:**

Here we present T-CUP 2.0, a model for predicting co-receptor tropism. Based on our recently published T-CUP model, we developed a more accurate and even faster solution. Similarly to its predecessor, T-CUP 2.0 models co-receptor tropism using information of the electrostatic potential and hydrophobicity of V3-loops. However, extracting this information from a simplified structural vacuum-model leads to more accurate and faster predictions. The area-under-the-ROC-curve (AUC) achieved with T-CUP 2.0 on the training set is 0.968±0.005 in a leave-one-patient-out cross-validation. When applied to an independent dataset, T-CUP 2.0 has an improved prediction accuracy of around 3% when compared to the original T-CUP.

**Conclusions:**

We found that it is possible to model co-receptor tropism in HIV-1 based on a simplified structure-based model of the V3 loop. In this way, genotypic prediction of co-receptor tropism is very accurate, fast and can be applied to large datasets derived from next-generation sequencing technologies. The reduced complexity of the electrostatic modeling makes T-CUP 2.0 independent from third-party software, making it easy to install and use.

## Background

The Human Immunodeficiency Virus 1 (HIV-1) enters host cells by binding to the CD4 receptor and one of the chemokine co-receptors CCR5 and CXCR4, [1]. The so-called co-receptor tropism of an HIV-1 virus describes the type of co-receptor that is being used: those viruses binding specifically to the CCR5 receptor are called "R5"-, and those binding to CXCR4 are called "X4"-tropic. Some viruses are able to bind either of the co-receptors and are called "dual"- or "R5X4"-tropic. It has been shown that patients harboring X4-tropic viruses tend to progess faster towards the Aquired Immunodeficiency Syndrome (AIDS) when compared to patients harboring only R5-tropic viruses [2]. Recently developed drugs, such as Maraviroc [3] and Vicriviroc [4], specificially bind to the CCR5 receptor, effectively inhibiting viral entry of R5-tropic viruses. Unfortunately, these drugs are of course ineffective against X4-tropic viruses. Therefore reliable determination of co-receptor tropism is crucial for an effective antiviral treatment of patients. Research has focused on the development of both *in vitro* tests, such as cell-based assays on the one hand, and *in silico* methods, on the other to develop a reliable tool for co-receptor tropism determination. The main drawbacks of the former are rather high costs and long turn-around time.

Most of the computational methods focus on the third variable loop (V3), a variable region of the glycoprotein 120 (gp120) of HIV-1. V3 is around 35 amino acids in length, variable in its sequence composition but also in length, and has been shown to be the main determinant for co-receptor tropism [5]. Electrostatic interactions have been implicated to play a decisive role in co-receptor tropism. The most simple and best-known model of co-receptor usage is the 11/25 rule [6] predicting a virus to be R5-tropic unless one of the amino acids sidechains at position 11 or 25 is positively charged. Although having a high specificity (about 90%), this rule lacks sensitivity (40-60%). In order to improve prediction accuracy, several more sophisticated prediction models, ranging from artificial neural networks [7], position specific scoring matrices [8] to support vector machines [9] have been developed. In our recent studies, we have taken up the implication made by charge-based rules (11/25) and developed an electrostatic hull approach to predict co-receptor tropism [10]. In our approach, V3 sequences (from the training set as well as sequences from new patients) are first modeled onto the V3 X-ray structure by Huang *et al.*[11] (PDB:2b4c). Second, the electrostatic potential *ϕ*(*r*) around the V3 loops is estimated by calculating the electrostatic potential on a constant hull, a discretized surface of *n*_*hull*_ = 642 points in an approximate distance of 0.6 nm around the solvent accessible surface of the V3 structure. The electrostatic hulls of the V3 loops are then used as input for a random forest model [12]. In addition, we complemented the structural electrostatic descriptor with a sequence-based classifier, the hydrophobicity scale by Kyte and Doolittle [13], which encodes amino acid sequences with numerical values representing their hydrophobicity. The hydrophobicity descriptor has been used in several other studies and a wide range of applications leading to accurate predictions [14-19]. The electrostatic and hydrophobicity classifiers, are then combined by a second-level-learning approach [20], i.e. the outputs of the two classifiers are used as an input for a third random forest, making the final prediction. With this scheme, we outperformed the prediction accuracy of other state-of-the-art methods, such as geno2pheno [21] and wetcat [9], as demonstrated on an independent test set [10]. Introducing structural information into classification models seem to improve overall prediction performance [10,22-24]. The combination of structural and sequence information has not only been demonstrated in HIV-1 co-receptor usage prediction, but also in other related studies dealing with HIV-1 drug resistance prediction [25,26]. For instance, Hou *et al.* developed an SVM-based method that modeled HIV-1 protease inhibitor resistance using structural information of the HIV-1 protease [25]. We proposed a classification model for Bevirimat resistance in HIV-1 that combines sequence-derived and structural information of the viral p2 protein [26]. These studies suggest that the combination of sequence and structural information can improve prediction performance, compared to classifiers based on either sequences or structures. This is in line with theoretical findings that ensemble learning can lead to better prediction results and that classifier diversity is highly important [27]. The rather complex modeling and prediction scheme of our initial co-receptor prediction method (called T-CUP) leads to disadvantages in computation speed, and involves a handful of external programs. Thus, to date T-CUP has not been available. The aim of this study was the development of T-CUP 2.0, a less complex and faster method, that yields better or comparable predictive power and is easy to install and to use.

## Methods

### Data

For this study, we used the data gathered by Dybowski *et al.*[10]. It consists of 1351 clonal amino acid sequences of the V3 loop of HIV-1 from 899 patients. 200 sequences are derived from X4-tropic (34 R5X4, 166 X4) viruses and 1151 from R5-tropic viruses. Most of the sequences are from subtype B (~52%), subtype C (~17%) and subtype D (~9%). However, 22% of the sequences spread over many different subtypes. Sequences, tropism and subtype information was extracted from the Los Alamos HIV database ( http://www.hiv.lanl.gov/).

### Sequence Interpolation

All sequences were transformed to a uniform length of 35 and encoded by the numerical hydropathy descriptor using the Interpol package [28] of R[29]. Interpol uses a numerical representation of the amino acids (here: hydropathy) and concatenates these data points. From the resulting curve samples are taken by equal interval. These samples are then used as features for classification.

Linear interpolation on a set of data points (*x*_0_,*y*_0_),(*x*_1_,*y*_1_),…,(*x*_*n*_,*y*_*n*_) is defined as the concatenation of linear interpolants between each pair of successive data points. The *normalization factor* is defined as the number of samples taken (by equal interval) from the aforementioned curves to generate an input for the subsequent classification. Here we used a normalization factor of 35.

### Clustering

In the V3 model, the datapoints were clustered using the k-Means algorithm in R. The number of centers was set to 35.

### Machine Learning

Random forests [12] were used for developing a classification model, as implemented in the randomForest package [30] of R. Receiver operating characteristics (ROC) curves were calculated and analyzed using the ROCR package [31]. Cross-validation was performed based on a leave-one-patient-out scheme according to Dybowski *et al.*[10], where the random forest was trained on all sequences of all patients except one patient, and the tropisms of the sequences of the remaining patient were predicted. This was repeated for each patient. This patient-wise-cross-validation was repeated for 10 times to average prediction performance. Area under the curve (AUC) values are shown as *a* ± *δ*, marking the average and the 95% confidence interval estimated with a t-distribution. Feature importance was assessed using the built-in function of the randomForest package and estimated by the *sum of all decreases in Gini impurity*, which has been shown to be more robust compared to the *mean decrease in accuracy*[32]. For statistical comparison we used Wilcoxon signed-rank test on the AUC distributions.

### Diversity

Classifier diversity has been shown to play an important role in classifier ensembles. Thus, we calculated classifier diversity in two ways. First, diversity was calculated based on the Spearman correlation between the classifier outputs *o*^*i*^ and *o*^*j*^ of classifier *i* and *j* for all samples *k* ∈ {1,…,*n*}. Second, we measured the disagreement of classifier *i* and *j*, i.e.

(1) $$D_{i,j}=\frac{1}{n}\cdot \overset{n}{\underset{k=1}{\sum }}|o_{k}^{i}-o_{k}^{j}|$$

### Comparison with other methods

For comparison with other prediction methods, we used the independent testset of Dybowski *et al.*[10]. This testset consists of 74 sequences from different HIV-1 subtypes. We compared T-CUP 2.0 with geno2pheno [21], wetcat [9] and the recently developed method of Bozek *et al.*[23].

## Results and discussion

### Overall approach

#### V3 Model

The motivation for a new prediction model for HIV-1 co-receptor usage is that T-CUP has a very high accuracy, but is rather slow due to the modeling process of the V3 loops with Modeller [33] and the calculation of the electrostatic hull with APBS [34]. This is especially important for new sequencing technologies, i.e. next-generation sequencing, where millions of sequences are generated per sample and fast predictions are needed to be applicable in routine diagnostics. Albeit T-CUP has been demonstrated to be applicable to next-generation sequencing data [35] in principle, it lacks computational efficiency. Therefore, we decided to develop a novel structural descriptor that is both, highly accurate with regard to subsequent classification, but also very efficient with regard to computing time.

The original T-CUP electrostatic classifier was based on an ESP hull above the V3 loop surface. Discretized values of the potential were used to predict co-receptor usage. However the calculation of these electrostatic potential values required solving the Poisson-Boltzmann equation (PBE), which is computationally expensive. The discretized ESP values are the result of charges found on the V3 loop and change when different values for the permittivity of these charges are assumed. In our original publication, we tested different values for permittivity for the solvent and protein. We found that the T-CUP electrostatic model has a good accuracy for a dielectric constant of 5 inside and outside the protein and an ionic strength of zero. Although these values do not reflect physiological conditions, the prediction accuracy was best. For T-CUP 2.0, we exploit the fact that under these conditions (equal permittivity of solvent and protein) the Poisson-Boltzmann equation can be reduced to a potential based on Coulomb’s law.

In our new model, in the following referred to as T-CUP 2.0, coordinates of the *Cα* atoms of the V3 structure of Huang *et al.*[11] were used as the basis for model building. First, V3 sequences were encoded with Interpol [28] using the net charge descriptor [36] and transformed to 35 values. For each sequence, the interpolated charge values were assigned to the template *Cα* coordinates. In this way, we can simplify the solution for the NP-hard side-chain packing problem [37] faced during the modeling step of the original T-CUP [10]. Next, the V3 structure was placed into a three-dimensional grid with a spacing of 1. Grid points that lay within a distance of [5,6] to any of the *Cα* were extracted. The resulting 8372 points were reduced by *k*-Means clustering (*k* = 35). Here, *k* was set to match the number of *Cα* atoms present in the model and thus serving as cluster centers. In the last step, the electrostatic potential (*ϕ*(*x*_*i*_)) for each of the cluster centers *x*_*i*_ was calculated by a vacuum model.

Our idea is based on our findings [10] that the T-CUP electrostatic model has a good accuracy for a dielectric constant of 5 inside and outside the protein and an ionic strength of zero. Under these conditions, the Poisson-Boltzmann equation can be reduced to a potential based on Coulomb’s law.

(2) $$\phi (x_{i})=\frac{1}{4\pi \varepsilon_{0}\varepsilon }\overset{n}{\underset{j\neq i}{\sum }}\frac{q_{j}}{r_{\text{ij}}}.$$

For the classification, we neglect all constant factors

(3) $$\phi {\left(x_{i}\right)}^{\prime }=\overset{n}{\underset{j\neq i}{\sum }}\frac{q_{j}}{r_{\text{ij}}}$$

The potentials for all *x*_*i*_ in the vacuum model were calculated by

(4) $$\phi (x_{i})=\overset{n}{\underset{j=1}{\sum }}\frac{q(C\alpha_{j})}{d(x_{i},C\alpha_{j})}$$

The resulting *ϕ*(*x*_*i*_) values were then used as a descriptor for the classifier, similar to the ESP values in the former model [10].

#### Second-level classification

Besides the electrostatic vacuum model, we also trained a classifier using the hydropathy scale according to Dybowski *et al.*[10]. The V3 sequences were encoded and interpolated to a length of 35 using Interpol [38]. The outputs of the vacuum model and the hydropathy model were then combined via stacking according to Dybowski *et al.*[10].

### Performance

**Figure 1:**
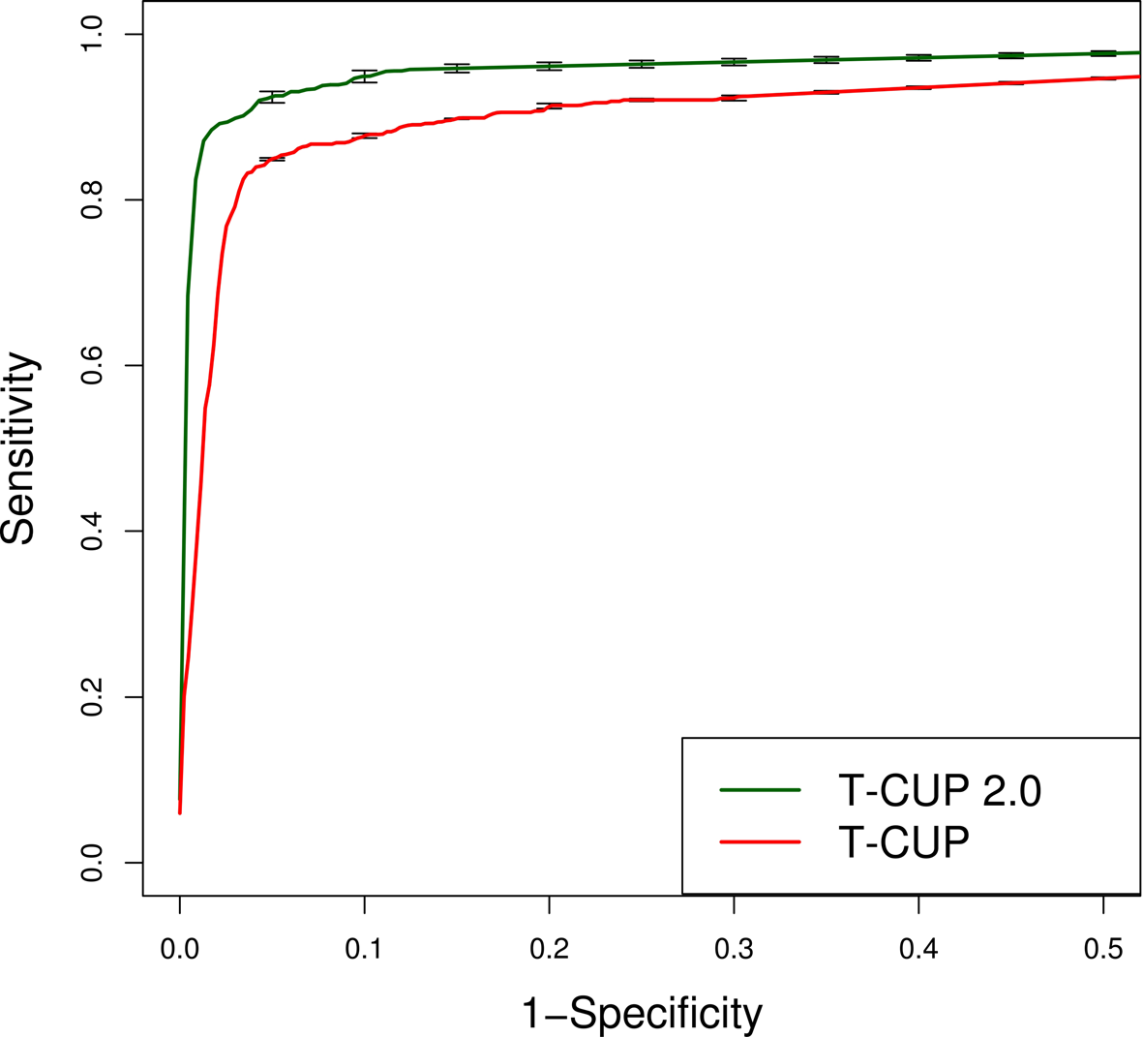
**ROC curves.** Comparison of T-CUP [10] (red) and T-CUP 2.0 (green) based on the patient-wise-cross- validation.

Using a patient-wise cross-validation scheme (see Methods) we demonstrated that T-CUP 2.0 performs superior compared to T-CUP with regard to prediction performance. ROC curves shown in Figure 1 show that T-CUP reaches an AUC of 0.937 ± 0.004 and is surpassed by T-CUP 2.0 with AUC of 0.968 ± 0.005 (*p* < 0.0001). We additionally evaluated the performance on an independent test set that has been compiled from literature by Dybowski *et al.*[10] and consists of 74 V3 sequences, including R5-, X4- and R5X4-tropic viruses. T-CUP 2.0 outperformed T-CUP also on this test set as shown in Table 1 (We calculated the sensitivities at certain specificities for the different algorithms). For instance, T-CUP 2.0 reaches a sensitivity of 85% at a specificity of 95% (i.e. a false positive rate of 5%), whereas T-CUP only achieves a sensitivity of 73% at a specificity of 95%.

**Table 1:** Comparison of different prediction methods

**Method**	**Sensitivity**	**Specificity**
geno2pheno	0.31	0.98
wetcat	0.63	0.98
T-CUP	0.68	0.98
T-CUP 2.0	0.70	0.98
Bozek et al.*	0.75*	0.96*
T-CUP	0.73	0.95
Bozek et al.	0.81	0.95
T-CUP 2.0	0.85	0.95
T-CUP	0.81	0.83
Bozek et al.	0.94	0.83
T-CUP 2.0	0.95	0.83

Methods are evaluated on an independent test set of 74 sequences collected from the literature with experimentally validated tropism. *:The method of Bozek et al. does only provide results for a specificity ≤ 0.96.

### Importance analysis

**Figure 2:**
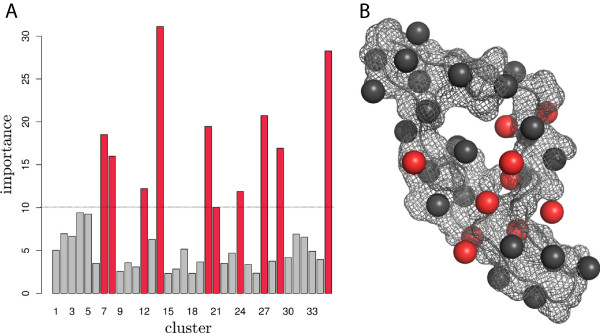
**Importance analysis. A**: Importance for each cluster for the classification process, measured as sum of all decreases in Gini impurity. Highly important clusters (importance >10) are colored in red. **B**: Clusters are shown as spheres in the V3 crystal structure. Highly important clusters (importance >10) are shown as red spheres.

Besides producing accurate predictions, random forests can also be used to estimate the importance of features used in the model. To this end, we calculated the *sum of alldecreases in Gini impurity* for each cluster in the vacuum model. The results are shown in Figure 2. The most important clusters (importance >10) in the vacuum model are 7, 8, 12, 14, 20, 21, 24, 27, 29 and 35. These positions are close to sequence positions 11 and 25 in the V3 structure and hence are in line with the findings from Dybowski *et al.*[10], who also predicted the most important regions of the electrostatic hull to be around these positions. Furthermore, these findings agree with the 11/25 rule. The clusters are not exactly at position 11 and 25 due to the modeling process, as they represent grid points and not the atoms in the structure.

### Diversity analysis

**Figure 3:**
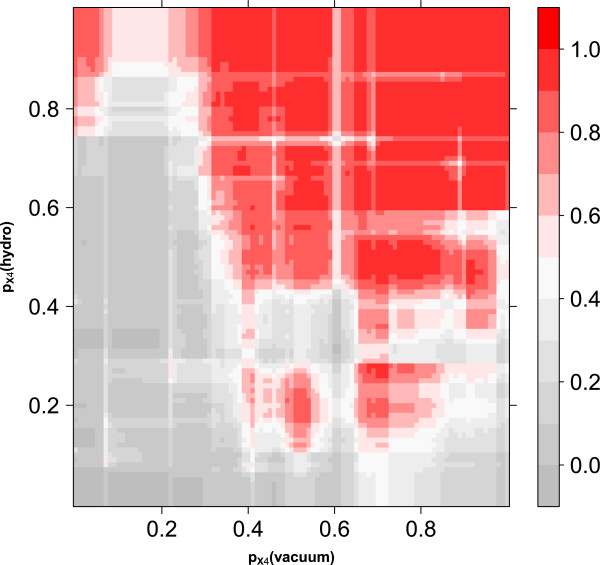
**Prediction landscape of T-CUP 2.0.** On the x-axis the prediction probability for a virus being X4 for the vacuum model is shown. On the y-axis the corresponding probability of the hydropathy model is shown. The colorscale shows the final X4-probability of T-CUP 2.0. The more red, the higher the X4-probability.

We then measured the diversity of the two first level classifiers, namely the vacuum and the hydropathy model in two ways: We calculated Spearman’s rank correlation coefficient *ρ* of the predicted X4-probabilities of the training samples, and the disagreement of the two classifiers. The correlation of the two classifiers in T-CUP 2.0 is *ρ* = 0.6381 ± 0.0009. For comparison, the correlation of T-CUP, namely the correlation of the ESP and the hydropathy classifiers is *ρ* = 0.6096. The disagreement measure in T-CUP 2.0 is *D* = 0.0662 ± 0.0003 and 0.0752 in T-CUP. Thus, the diversity is slightly higher in T-CUP compared to T-CUP 2.0. This is visualized in the prediction landscape for T-CUP 2.0 as shown in Figure 3. The fact that the diversity of T-CUP is slightly higher compared to T-CUP 2.0 is unexpected as the overall performance of T-CUP 2.0 is higher. In contrast to the assumption that a higher diversity within a classifier ensemble leads to a better prediction, T-CUP 2.0 demonstrates the opposite. Nevertheless, the diversity is only slightly decreased and this seems to have no negative effect to the prediction accuracy.

### Template analysis

We took V3 loops from two X-ray structures as templates, both with V3 in the context of CD4 bound gp120 and an antibody, to test whether the template structure of the V3 loop has an impact on the classification performance. First the V3 structure from PDB entry 2b4c [11] was used, based on the R5-tropic strain JR-FL and bound to antibody X5. For the second round we used V3 from PDB entry 2qad [39], based on R5-tropic strain YU2 and complexed with the sulfated antibody 412d. The latter V3 structure is less open in the central bulge region of V3 as it there binds one of the sulfate groups. Both V3 sequences have a length of 35 amino acids and carry only three conservative mutations. Surprisingly, using a more recent V3 structure [39], we found no significant differences in AUC distributions according to the Wilcoxon signed-rank test (*p* = 0.25). Moreover, we also tested a model that, besides hydrophobicity and charge, employs the length of the V3 sequences. This is motivated by the notion that the sequence length in the training set is rather narrowly distributed in R5-tropic (83% have length of 35 amino acids), while being broader in X4-tropic viruses (only 52.5% have length of 35 amino acids). However, incorporating length information into the classification system did not improve classification performance (Wilcoxon signed-rank test *p* = 0.44).

### Evaluation on NGS data

Both algorithms, T-CUP and T-CUP 2.0 have a linear computational complexity (in *O*(*n*)). T-CUP 2.0 is completely written in R and up to 14-times faster than the original T-CUP. For data resulting from a 454/Roche GS FLX sequencing run and consisting of around 1 million reads, T-CUP 2.0 needs 3.1 days on a single CPU, while T-CUP requires 43.7 days. Both systems can be run in parallel, leading to around 9 hours for T-CUP 2.0 (two Quadcore server with IntelXeon(R) CPU E5462 @ 2.80GHz and 32GB RAM), and five days for T-CUP still.

**Figure 4:**
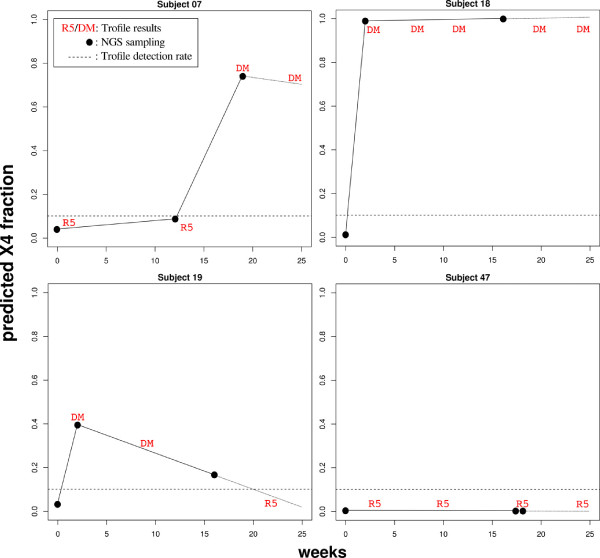
**Development of X4-fraction.** Development of predicted fraction of X4-using viruses during treatment. Labels "R5" (R5-using) and "DM" (dual/mixed or X4-using) are Trofile results at the given times. The dotted line marks the 10% detection rate of standard Trofile assay. The black dots represent the time points when the NGS data was sampled, the solid lines are used for visualization of the fraction of X4-using viruses between these measured data points.

We also evaluated the performance with real next-generation sequencing data taken from Tsibris *et al.*[40] and compared the prediction of T-CUP 2.0 with the results from phenotypic assays. The next-generation sequencing data was generated from samples of four patients at three timepoints during treatment with the co-receptor antagonist Vicriviroc. Additionally, phenotypic tropism predictions were acquired at several timepoints. All patients had R5-tropic viruses at treatment start, but experiences therapy failure after a few weeks into treatment. We extracted the V3 sequence from the next-generation sequencing reads according to Dybowski *et al.*[35] and predicted the X4-fraction of each time point with T-CUP 2.0. As for the old model T-CUP [35], the predicted X4-fractions are perfectly in line with the results from the phenotypic assays (see Figure 4).

## Conclusions

T-CUP 2.0 performs superior compared to T-CUP in both, the patient-wise cross-validation and on the independent test set. When comparing prediction results between T-CUP 2.0 and T-CUP the correlation is relatively high *r* = 0.702 ± 0.001. The disagreement *D* = 0.1001 ± 0.0014, indicates that classifiers can be combined into a final, more accurate prediction. Also Chueca *et al.*[41] have suggested to combine different prediction models to improve overall prediction performance. Additionally, we have demonstrated the use of our method with next-generation sequencing data. The predicted X4-fraction is in agreement with results obtained from phenotypic assays. However, computational method such as T-CUP 2.0 are able to detect smaller fractions of X4-viruses compared to current phenotypic assays and thus should be considered as more sensitive for diagnostics issues with next-generation sequencing data [42]. Finally, we are convinced that making the T-CUP 2.0 R-package available to other researchers is a valuable contribution to the field.

## Availability and requirements

**Project name:** T-CUP 2.0

**Project home page:**http://www.uni-due.de/~hy0546/TCUP2.zip

**Operating system(s):** cross-platform (64bit architecture)

**Programming language:** R

**Other requirements:** R 3.0 or higher

**License:** GNU LGPL

**Any restrictions:** none

## Competing interests

The authors declare that they have no competing interests.

## Authors’ contributions

Conceived and designed the experiments: DoHe, JND, DaHo. Performed the experiments: DoHe. Interpreted results: DoHe, JND, CW, DaHo. Wrote the paper: DoHe. All authors read and approved the final manuscript.
